# OncoPDSS: an evidence-based clinical decision support system for oncology pharmacotherapy at the individual level

**DOI:** 10.1186/s12885-020-07221-5

**Published:** 2020-08-08

**Authors:** Quan Xu, Jin-Cheng Zhai, Cai-Qin Huo, Yang Li, Xue-Jiao Dong, Dong-Fang Li, Ru-Dan Huang, Chuang Shen, Yu-Jun Chang, Xi-Ling Zeng, Fan-Lin Meng, Fang Yang, Wan-Ling Zhang, Sheng-Nan Zhang, Yi-Ming Zhou, Zhi Zhang

**Affiliations:** 1 National Engineering Research Center for Beijing Biochip Technology, Changping District, Beijing, 102206 P.R. China; 2 CapitalBio Corporation, Changping District, Beijing, 102206 P.R. China; 3 School of Medicine, Tsinghua University, Beijing, 100084 P.R. China; 4 College of Marine Life Sciences, Ocean University of China, Qingdao, 266003 P.R. China

**Keywords:** Oncology pharmacotherapy, Alterations, Implications, Drug indications, Clinical trials, Clinical support tool, Knowledgebase

## Abstract

**Background:**

Precision oncology pharmacotherapy relies on precise patient-specific alterations that impact drug responses. Due to rapid advances in clinical tumor sequencing, an urgent need exists for a clinical support tool that automatically interprets sequencing results based on a structured knowledge base of alteration events associated with clinical implications.

**Results:**

Here, we introduced the Oncology Pharmacotherapy Decision Support System (OncoPDSS), a web server that systematically annotates the effects of alterations on drug responses. The platform integrates actionable evidence from several well-known resources, distills drug indications from anti-cancer drug labels, and extracts cancer clinical trial data from the ClinicalTrials.gov database. A therapy-centric classification strategy was used to identify potentially effective and non-effective pharmacotherapies from user-uploaded alterations of multi-omics based on integrative evidence. For each potentially effective therapy, clinical trials with faculty information were listed to help patients and their health care providers find the most suitable one.

**Conclusions:**

OncoPDSS can serve as both an integrative knowledge base on cancer precision medicine, as well as a clinical decision support system for cancer researchers and clinical oncologists. It receives multi-omics alterations as input and interprets them into pharmacotherapy-centered information, thus helping clinicians to make clinical pharmacotherapy decisions. The OncoPDSS web server is freely accessible at https://oncopdss.capitalbiobigdata.com.

## Background

Oncology pharmacotherapy focuses on treating cancer with drugs rather than other cancer therapies such as radiotherapy and cytotherapy. In addition to the rapid progress that has been made towards understanding the genetic heterogeneity and mutational landscape underlying cancer, significant advances have been made in oncology pharmacotherapy. Specifically, in recent years, large number of efficacious drugs have been developed that have greatly improved the survival time of cancer patients. Many scientists have attempted to match drugs to the right patients using evidence-based information about individual somatic mutations and structural alterations present in patient tumors. Information discriminating whether an alteration is clinically actionable resides in various silos such as US Food and Drug Administration (FDA) labeling, National Comprehensive Cancer Network (NCCN) guidelines, expert group recommendations, and the scientific literature. As only a small subset of alterations identified from the whole genome, whole exome or DNA methylation sequencing are driver mutations that are clinically actionable [1], it is necessary to ensure that these actionable alterations are recorded in the knowledge base; otherwise patients may receive genetic tests but receive little or no pharmacotherapy suggestions. An increasing number of studies have revealed the importance of epigenome as well as other omics factors in explaining the influence on drug responses, especially in anti-cancer drug responses [2–5], thereby incorporating genomic, epigenomic, and other omics technologies that may improve prediction accuracy of drug treatment responses. In addition, some pharmacotherapy drugs are still in the clinical trial stage and thus are not available to the public or have been approved by administration agencies but the prices are unaffordable for some cancer patients. In these situations, providing the most suitable clinical trials to those patients may be a better choice.

Recently, efforts have been made to establish a precision medicine knowledge base as well as a platform that synthesizes the interpretation of cancer genomes. Clinical Interpretations of Variants in Cancer (CIViC) provides a centralized curation interface for the community to develop a consensus by leveraging an interdisciplinary, international team of experts to collaborate remotely [6]. The Oncology Knowledge Base (OncoKB) is a comprehensive knowledgebase that offers precision oncology information to support optimal treatment decisions [1], and the Precision Medicine Knowledge Base (PMKB) is a structured database for clinical-grade mutations and interpretations [7]. All of these resources collect knowledge from various silos while only providing a single term query via web interface or application programming interface and cannot interpret the bulk of alterations in a single query. The Cancer Genome Interpreter (CGI) is a platform that automates the interpretation of the newly sequenced cancer genome to identify driver alterations and their possible effects on treatment response [8]. It also organizes evidence at different levels but does not specify which treatment may be effective based on the evidence. PanDrugs provide a bioinformatics platform that can be used to identify potentially druggable molecular alterations and prioritize anticancer drug treatments according to individual genomic data [9]. It realizes the goal of identifying drugs that can be considered when making a clinical decision but prioritizes drugs mainly from the genetic level rather than at the gene variant level. The Mutation to Cancer Therapy Scan is a tool that classifies drugs into three categories to describe the sensitivity of evidence-based drugs [10]. It only focuses on efficacy and does not include the toxicity information of each drug, nor does it take the approval status of the drugs into consideration.

To fulfill the urgent need to interpret genomic alterations in patients’ tumors, the Oncology Pharmacotherapy Decision Support System (OncoPDSS) web-based tool was developed to aid clinicians and oncologists with clinical pharmacotherapy decision making. This knowledgebase consists of multi-omics alterations’ actionable evidence and drug indications as well as information on cancer clinical trials. A pharmacotherapy centric classification system was also developed to decipher the effectiveness and safety of each therapy based on the constructed knowledgebase. OncoPDSS accepts individual-level genetic test results as input, followed by matching to alterations in the knowledgebase to retrieve all related evidence, and are then used to classify the pharmacotherapies. OncoPDSS bridges the communication of laboratory tests and clinical decision making, helping researchers and clinicians interpret the actionable meaning of each detected alteration at the individual level.

## Implementation

### Database of pharmacotherapy evidence

**Fig. 1:**
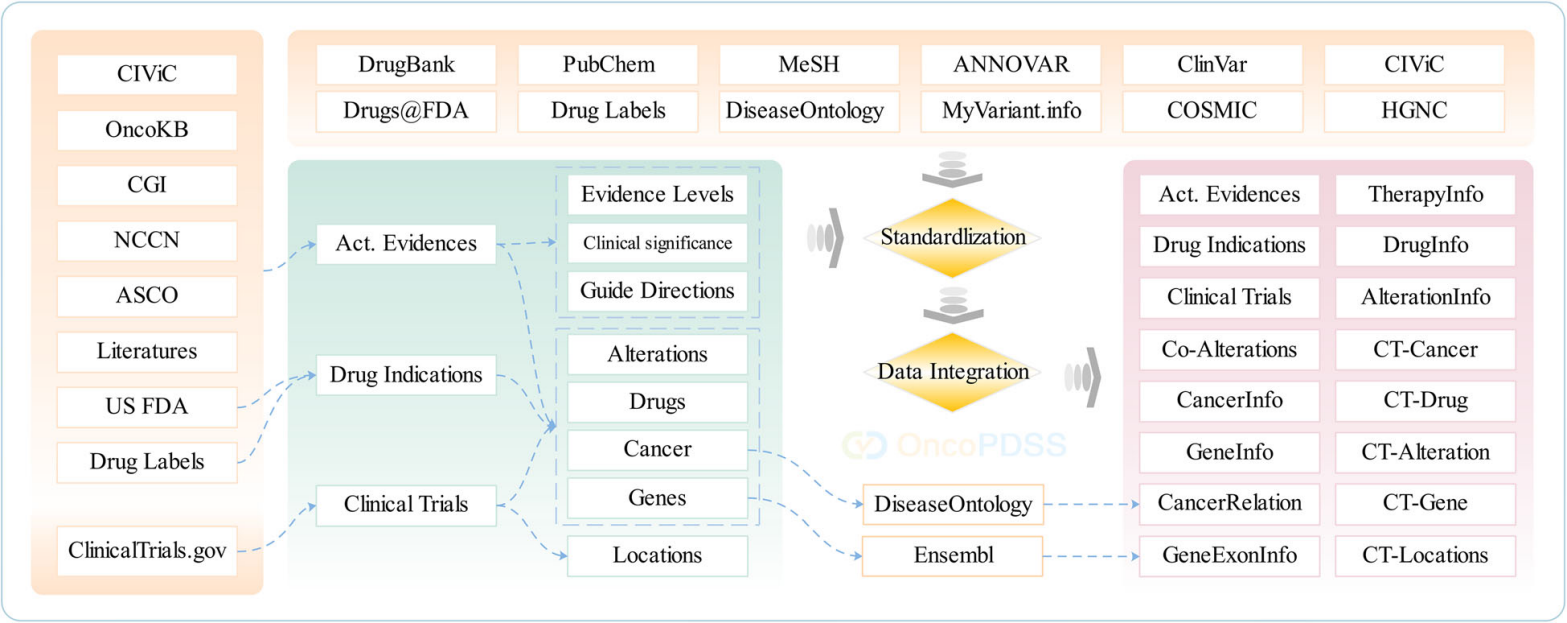
OncoPDSS knowledge base (OncoPDSSkb). OncoPDSSkb integrates three types of evidence from several public resources, and the elements in these datasets are standardized under public standards

The OncoPDSS knowledgebase (OncoPDSSkb) has been constructed to record oncology pharmacotherapy evidence (Fig. 1). Alteration-drug associations or so-called actionable evidence were collected from the CIViC, CGI, OncoKB and PubMed databases. OncoKB curates clinically actionable alterations from FDA labeling, NCCN guidelines, and other resources; CIViC includes cancer variants from public studies and is subjected to rapid updates; and CGI includes information on the influence of genomic alterations to drug responses. Together, these databases collect most of the currently available actionable evidence, and with our own curation of alterations garnered from hundreds of scientific papers, a comprehensive actionable alteration database was constructed. In addition, cancer clinical trials and FDA-approved drug indications were also curated and integrated into the OncoPDSSkb. The cancer clinical trials data were downloaded from the ClinicalTrials.gov database in XML format [11], the cancer and pharmacotherapies of each clinical trial were retrieved directly from the data fields of the XML file while the gene and alterations were curated by keyword matching followed with manually audit. The drug indications were manually curated from drug labels that were downloaded from the Drugs@FDA database [12]. All these data consist of pharmacotherapy evidence.

### Standardization, updating, and versioning

To better integrate the evidence from different sources, cancer, alterations, genes and drugs were extracted and standardized. Disease ontology was introduced as a standard cancer term [13], and all cancer names from the evidence were exactly matched to the disease ontology term. Several cancer types such as “any solid tumor” were directly added to the cancer tree as a child node of “non-disease ontology terms” (see Additional file 1, Table S1). In addition to standard names, the hierarchical relationships of each term were also retrieved from disease ontology. The HUGO Gene Nomenclature Committee gene symbol was used as the standard of each gene. Gene exon region data were queried and retrieved from the Ensembl database [14, 15]. The alterations were first annotated by MyVariant.info [16], followed by duplication removal and data integration. The multi-omics alterations were classified into 29 types in four categories (see Additional file 1, Table S2). The inclusion relationship among different alterations were labeled as “is-A.” For example, EGFR:L858R is-A EGFR:Activating Mutations, as well as EGFR:exon21mut and EGFR:Mutations. The anti-cancer agents were integrated based on the generic name, brand name, chemical name, CAS number, and other synonyms. The generic name for each approved drug or the R&D code for each developing agent served as the standard drug name. To help cancer researchers and clinical oncologists better use the platform and understand the interpretation reports, plenty of annotation information on the abovementioned ingredients were retrieved from various silos (see Additional file 1, Table S3). The location information of the clinical trials was also extracted from the ClinicalTrials.gov database.

To take into consideration the evidence levels in the following interpretation step, another standardization was done. All actionable evidence was classified into six categories, namely case reports, clinical trials, approved drug labels, guidelines, inferential procedures, and pre-clinical information according to their initial sources (see Additional file 1, Table S4). In addition, the clinical significance and direction of each evidence were used to help the interpreter “understand” the meaning of the responses change of alterations on the pharmacotherapy under certain cancer type(s). Five types of clinical significance related to three guide directions were concluded (see Additional file 1, Table S5). “Positive” means the therapy can be considered under the current type of cancer with certain alteration/co-alterations by considering the efficacy or toxicity; “negative” has the reverse meaning; and “uncertain” means that it is unclear if the therapy is safe or effective.

As new laboratory and clinical data are continually generated, the information on clinical trials may change over time. FDA labels and professional guidelines are also updated at irregular intervals, which may influence the pharmacotherapy choices of patients. To minimize the gap between OncoPDSSkb and community research findings or clinical consensus, OncoPDSS will regularly download and integrate actionable evidence and clinical trials data from the abovementioned resources, and will manually curate drug indications and actionable evidence from drug labels, guidelines, and scientific literature continuously. Because the interpretation results can be influenced with the update of knowledge, it is of great importance to label the database version for each report, thereby helping to keep the interpretation results traceable.

### Annotation, interpretation, and classification

**Fig. 2:**
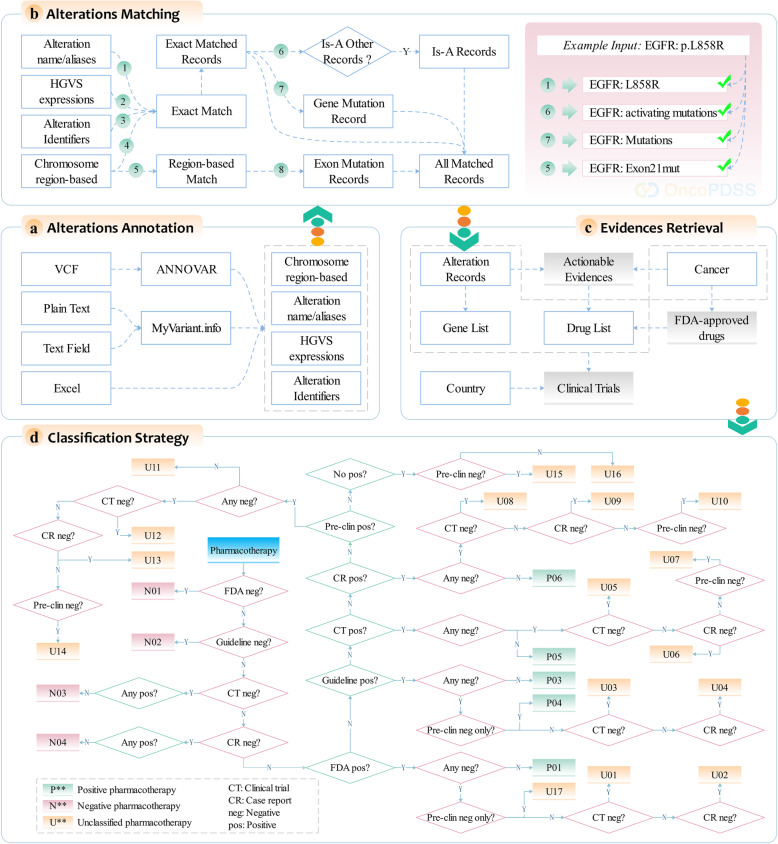
OncoPDSS workflow. **a** Alteration annotations. Different types of alterations undergo different annotations. **b** Alteration matching. The annotated alterations are subjected to eight kinds of processes to match to the alteration records. **c** Evidence retrieval. The matched alteration records will serve as one of the conditions to retrieve pharmacotherapy evidence. **d** Based on the retrieved evidence in the prior step, the drugs are classified into three categories under the classification strategy

Based on the integrated knowledgebase, OncoPDSS is a novel method for annotating and interpreting user-submitted alterations, summarizing the efficacy or toxicity risk of each pharmacotherapy, and provide related clinical trials for the potentially effective ones (Fig. 2). OncoPDSS takes alteration lists as input, either in variant call format (VCF), Excel, or plain text format. OncoPDSS assumes that quality controls have been previously applied and that alterations are of high quality. Different formats of alterations are subjected to different post-submit processes. ANNOVAR is used to efficiently convert VCF format alterations to Human Genome Variation Society format to better match the alterations to OncoPDSSkb alteration records (KBARs). Plain text format alterations are subjected to annotations by MyVariant.info to expand their descriptors under the same reason. As Excel format is a pre-defined alteration format and each field is well defined in accordance with the KBARs, the alterations do not need any tool for annotation before matching to the records (Fig. 2a). As plain text and Excel format support many more alteration types than VCF, such as transcriptome and epigenome level alterations, they are the recommended formats. User-uploaded alteration expression and the annotated results from either ANNOVAR or MyVariant.info will be directly matched to the KBARs under the exact match mode. In addition to the exact match of each alteration, OncoPDSS also collects the is-A alterations of each matched alteration. If an alteration has been matched, its “is-A” record(s) will also be retrieved. OncoPDSS also checks the regions for each insertion or deletion; for example, a deletion can be matched to an EGFR:exon19del record when both the first and last base is located in the exon 19 region of EGFR (Fig. 2b).

The cancer types are required to be selected before the interpretation. The selected cancer types (OncoPDSSkb cancer records, KBCRs) can be used to retrieve FDA approved drugs, and together with the matched KBARs can retrieve the actionable evidence. From both the drug indications and actionable evidence, a list of anti-cancer agents (OncoPDSSkb drug records, KBDRs), which may include FDA-approved drugs and clinical trial compounds, can be distilled (Fig. 2c). If a single actionable evidence contains more than one alteration or so-called co-alterations, it is required that all of these alterations be matched so that the evidence can be retrieved. Once the co-alteration evidence related to a certain therapy has been retrieved, the evidence related to each component alteration for the same therapy should be discarded. As the drugs in each evidence may come up as a single agent or combined with others—both of which were considered an independent pharmacotherapy—OncoPDSS reports the interpretation results as therapy centric, meaning that the evidence related to the combined therapies do not belong to any of their composition drug, and vice versa. Both the classification strategy and scoring method are made for these pharmacotherapies independently. For a certain pharmacotherapy, the FDA negative evidence is first checked, followed by guideline (e.g., NCCN, ASCO, ESMO) negative evidence, if exists, it is classified as negative therapy, otherwise the clinical trial and case report negative evidence is checked. If negative and nonpositive evidence exists, the therapy is also classified as negative. For a therapy to be classified as positive, one of the condition is that clinical-grade (FDA or guideline approved, clinical trial or case report proved) positive evidence exists while none negative evidence exists, another one is that FDA or guideline approved positive evidence and pre-clinical negative evidence co-exists. Beyond above mentioned conditions, the therapy is unclassified, which means whether the therapy is effective or not is not very sure, either with low level evidence or with conflict evidence. Different therapies may have their own evidence to be classified as one of the three categories, to distinguish the therapies from each other, the TScore is calculated for each therapy based on the evidence level and count:

$$ \mathrm{TScore}={\sum}_{i=1}^n\left({E}_i+{S}_{d\;i}\ast {C}_{d\;i}\right)+\lg \left({C}_{ct}+1\right) $$

where *n* is the total number of actionable evidence, *E*_*i*_ is the score of each actionable evidence that is assigned a confidence level of their resources (approved: 1, guideline: 0.8, clinical trial: 0.5, case report: 0.3, pre-clinical: 0.1, inferential: 0.1). If the actionable evidence is negative, the score is negative accordingly. *S*_*di*_ is the weighting factor (assigned as 0.5), *C*_*di*_ is the number of drug indication evidence, *C*_*ct*_ is the number of ongoing therapy-related clinical trials, and the number 1 in the logarithmic formula make sure the logarithmic calculation results are 0 when *C*_*ct*_ is 0 (see Additional file 1, Supplement on TScore equation). TScore is totally an evidence-based scoring method to show the evidence supporting status of each pharmacotherapy and based on which a sorted list of therapies under each category can be obtained. It must be noted that the sorted therapies do not directly reflect the effectiveness prioritization and is only one of the many ways to sort these therapies. In addition, the sorting step is done under each category, which means that it does not influence the classification results.

## Results

### OncoPDSSkb, a comprehensive oncology pharmacotherapy knowledgebase

At the time of writing (OncoPDSSkb version 1.0), 7692 actionable evidence, 526 drug indications, and 19,922 cancer related clinical trials are included in OncoPDSSkb. In addition, 2676 anti-cancer agents, 528 cancer or pharmacotherapy related genes, 372 cancer types and 13,889 alterations related to 29 alteration types are also included (see Additional file 1, Table S6).

### Web server design

OncoPDSS (version 1.0) is constructed on CentOS release 6.9 operating system and the datasets are managed by the MongoDB (version 3.4). Friendly interfaces are implemented by using Bootstrap, the dynamic functions are designed using the JavaEE technology (version 1.8) and run on a Tomcat server (version 8.0).

### Usage and interface

**Fig. 3:**
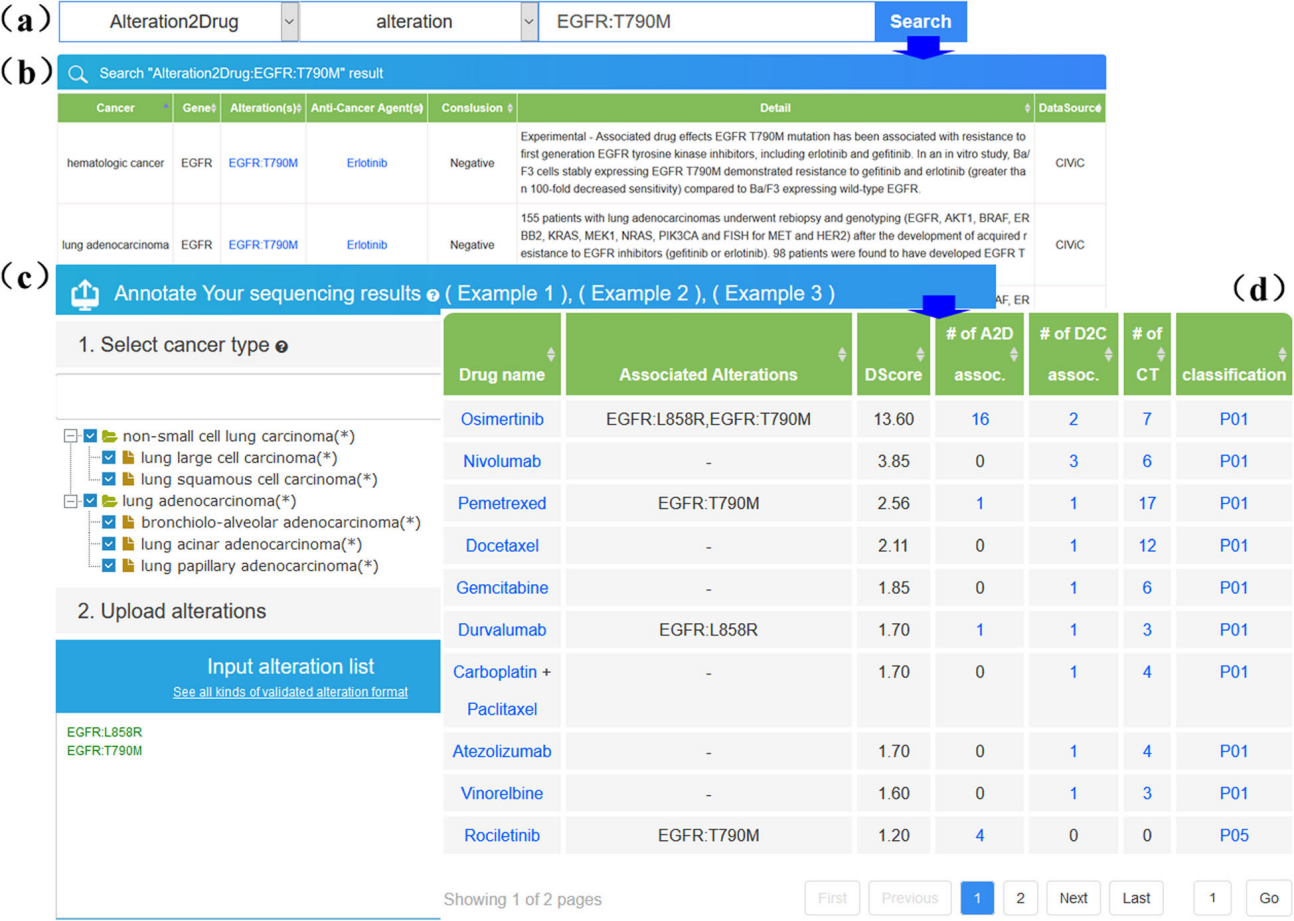
OncoPDSS web pages. **a** Query function, **b** Alteration-drug association query result, **c** Job creation page, **d** Positive pharmacotherapies in interpretation report

The OncoPDSS platform can be accessed through a simple and user-friendly interface at https://oncopdss.capitalbiobigdata.com. On clicking the “INTERPRET” button in the navigation bar, the user can be directed to the interpretation page. The cancer type is first selected via the cancer tree to investigate disease-specific evidence; one or more cancer types can be selected in an interpretation job. The closer the cancer types the user selects, the more meaningful the results. OncoPDSS accepts user-submitted alterations in standard VCF, pre-defined Excel or simple text file format, or input via the text field function (Fig. 3c). To best suit the personalized demands of diversified user groups, the “yes” or “no” options on drug indications and clinical trials information are provided. When the “no” option been selected, related evidence will not be retrieved and the final classification of pharmacotherapies will be influenced, as they will take actionable evidence into account only. To make the best of the system, the “yes” options are recommended and set as default. When the “yes” option of clinical trials is selected, the user is asked to do some other selections, such as the matching pattern, the country, and the status, thus helping to filter out the best suitable clinical trials for patients.

OncoPDSS displays the interpretation results as a web-based report in three sections. The job detail section shows the basic information of the current job, and the alteration matching results section lists the matching information for each actionable alteration. What is listed next is the drug classification result, which is further divided into three sub-sections, namely the “Positive pharmacotherapies” (Fig. 3d), “Negative pharmacotherapies”, and “Unclassified pharmacotherapies”. The “Positive pharmacotherapies” table lists all approved therapies with no negative evidence, and thus can be considered by the clinicians in the following decision-making process. The “Negative pharmacotherapies” table lists the therapies wherein at least one of their retrieved evidence indicated that it is negative and has been accepted by the FDA or guidelines. The “Unclassified pharmacotherapies” table lists the therapies with no authoritative evidence to classify them as positive or negative. For all therapies in these tables, actionable evidence is listed, and drug indications as well as clinical trials are also listed for the positive and unclassified categories, as both of them may be reviewed by the clinicians to consider whether any of the therapies is useful. In addition, to support single keyword query, OncoPDSS also provides a quick search function in the “QUERY” page (Fig. 3a). To start a query, the user is asked to choose the target dataset from the dropdown option list in the quick search form. Meanwhile, a complete or partial keyword is required to trigger the search. To demonstrate the usage of the query function, several sample keywords with hyperlinks are listed under the quick search form. When clicking each one of them, the dropdown menu will be selected, and the text box will be filled with a keyword. On clicking the “Query” button, all matched items in the target dataset will be listed in the search results (Fig. 3b).

### Usability evaluation

To evaluate the usefulness and effectiveness, several groups of actionable alterations were used to describe the feasibility of the OncoPDSS system. Non-small cell lung cancer (NSCLC) is the most common type of lung cancer, and the most common types of NSCLC are squamous cell carcinoma, large cell carcinoma, and adenocarcinoma [17]. EGFR is one of the driver genes in NSCLC; the most common activating mutations in EGFR are exon 19 deletion and L858R point mutation, which account for 80% ~ 90% of all EGFR mutations [18]. When squamous cell carcinoma, large cell carcinoma, adenocarcinoma as well as non-small cell lung carcinoma were selected as cancer types, and EGFR L858R as input alteration, records including EGFR:L858R, EGFR:Activating Mutations, EGFR:Exon21mut, and EGFR:Mutations were matched, and 104 alteration-drug associations were retrieved. EGFR tyrosine kinase inhibitors (EGFR-TKIs) such as gefitinib, erlotinib, afatinib, osimertinib are listed as positive pharmacotherapies, and due to the abundant evidence of these drugs, their TScores are the highest, which causes these therapies to be sorted on the top of the list. During the treatment of EGFR-TKIs, most NSCLC patients may become drug resistant due to the development of T790M mutation in EGFR [19–21]. When considering both EGFR L858R and EGFR T790M as input alterations, EGFR:T790M and four aforementioned alteration records were matched, and 137 alteration-drug associations were retrieved. Osimertinib is still listed as positive and sorted in the front of the positive therapies. Afatinib, gefitinib and erlotinib are changed to the “negative” category, as there are guideline-level negative evidence indicating that the existence of EGFR:T790M, or the co-existence of EGFR:L858R and EGFR:T790M mutations, is associated with acquired resistance to these drugs. Furthermore, recent clinical studies revealed that the substitution mutation C797S in EGFR can affect the binding of TKIs to EGFR, which confers resistance to all third-generation EGFR-TKIs, including osimertinib [22]. When EGFR L858R, T790M, and C797S were input together, 139 alteration-drug associations related to EGFR:L858R, EGFR:Activating Mutations, EGFR:Exon21mut, EGFR:Mutations, EGFR:T790M and EGFR:C797S records were retrieved, and none of the above EGFR-TKIs was listed as positive pharmacotherapies. In addition to the aforementioned EGFR-TKIs, there are many other therapies, such as several chemotherapies and immune checkpoint inhibitors, listed in each category, which can greatly broaden the scope of pharmacotherapy consideration.

## Discussion

Cancer sequencing have witnessed the shift from single gene tests and small hotspot panels to larger gene panels, whole-genome, and whole-exome sequencing, as well as other omics sequencing such as RNA-seq and ChIP-seq. A variety of different kinds of alterations can be detected for a single sample, and each of these alteration types may contribute to a change in the drug response; for example, T790M point mutation in EGFR can lead to the resistance of first- and second-generation EGFR-TKIs in NSCLC cancer patients, BCR-ABL fusion is a predictive effect of imatinib in both acute lymphocytic leukemia and chronic myeloid leukemia patients, and MLH1 methylation is associated with resistance to treatment with oxaliplatin in patients with stomach cancer [23]. To comprehensively interpret the alterations and best support clinical decision making, a system that supports multi-omics alterations annotation is of great importance.

In addition to the alteration types, coexistence of different alterations may also affect drug responses. When existing separately, activating EGFR mutations are well defined predictive effects of EGFR-TKIs in NSCLC cancer patients, and while coexisting with KRAS mutations and ALK or ROS1 gene rearrangements, the EGFR-TKIs are predicted to be resistant [24]. Co-existence of alterations can alter the responses of drugs not only in different genes but also in the same gene. As Masuzawa et al. reported, for EGFR:G719S + EGFR:T790M or EGFR:L861Q + EGFR:T790M, the IC50 values of third-generation EGFR-TKIs, such as osimertinib and nazartinib, were around 100 nM, which was 10- to 100-fold higher than those for classic+T790M mutations [25]. In a clinical case study, ALK G1202R was identified in a patient with ALK-rearranged non-small cell lung cancer after the disease progressed while on alectinib therapy, suggesting that with coexistence of ALK G1202R or not, the choices of alectinib in ALK-rearranged non-small cell lung cancer patients are different [26]. These co-alteration evidence confirm the necessity of taking all detected alterations into consideration rather than a single alteration when making the pharmacotherapy decisions, meaning that decisions should be made at the individual level. It is worth mentioning that when the evidence of a certain alteration as single or co-existing with other alteration(s), the single alteration evidence are to be discarded; this explains why the number of retrieved evidence of EGFR:L858R & EGFR:T790M is less than the number of EGFR:L858R only in the usability evaluation part.

During the drug development process, a great number of pre-clinical compounds have failed to show reproducible effects on patient survival [27]. One of the strategies being used to overcome this situation is the use of a combination of drugs that rely on complementary mechanisms of antitumor activity and can be combined into a therapeutic regimen [28]. In a preclinical study, colorectal cancer cell lines harboring BRAF V600E demonstrated decreased response to vemurafenib while they showed inhibited survival under vemurafenib and cetuximab combination treatment in culture [29]. This shows the efficacy difference between the combination and single agent therapies: evidence related to one of the therapies cannot be shared with another one. To keep the effectiveness of the interpretation report, both monotherapy and combined therapy should be included, and both should be processed independently. OncoPDSS collected all actionable evidence, drug indications and clinical trials with pharmacotherapy-centric, as well as reporting the interpretation result as pharmacotherapy-centric.

Pharmacotherapy evidence, especially the actionable evidence are the key constitute of the knowledgebase. The quality of the evidence can influence the TScore or even the classification result. The exact clinical significance definition of each evidence is particularly important. As OncoPDSS included evidence on drug efficacy as well as toxicity, the clinical significance definition should indicate whether the therapy is sensitive or resistant and safe or with toxicity risk in the certain sub-type of cancer. Moreover, there are evidence that did not clearly indicate the response, and the clinical significance is uncertain. As to clinical action, the sensitive and/or safe therapies are positive and the resistant and/or with high toxicity risks are negative.

Oncology pharmacotherapy is an especially important therapeutic method to use in the treatment of cancer patients. The final treatment decision can only be made by the chief clinician, and any databases or clinical decision support systems are the tools to help provide as comprehensive and accurate information as possible for the health care providers. Approval by the FDA is a prerequisite for classifying a therapy as positive. All the therapies that are in the positive category have been approved by the FDA. As the drug indication evidence only records the information on drug-cancer associations, no biomarker included, another prerequisite that the therapy can be classified as positive is that there is no authoritative negative actionable evidence indicating the non-effectiveness of the therapy. If any authoritative negative actionable evidence exists (i.e., FDA-approved, or guideline-included evidence), the therapy is classified as negative, whether or not it has been approved. Beyond these two situations, therapies are designated as unclassified, as there is no authoritative evidence to prove the effectiveness or non-effectiveness of them. As the unclassified category includes either the potentially effective but currently not approved therapies, or potentially non-effective while currently not accepted by the FDA or guidelines, it is a great resource for benefit helping oncology scientists design successful clinical trials, as well as helping clinicians avoid the wrong use of non-effective therapies as far ahead as possible in clinical practice.

As for cancer patients, there are several things that they should take into consideration when choosing the best suitable pharmacotherapy, such as the efficacy, toxicity, and accessibility. Cancer patients are living longer lives from successful cancer treatments that are the results of past clinical trials. Through clinical trials, patients can receive access to effective as well as safe treatments much earlier, especially for cancer patients with no potentially effective therapy to select. Targeted therapy drugs as well as immunotherapy drugs bring great progress in efficacy, thus leading to a tremendous extension of both overall survival (OS) and progress free survival (PFS) of cancer patients as compared to traditional chemotherapy. Though effective, the prices for these therapies are much higher than for chemotherapies, and a segment of the patient population cannot afford them at all. In this circumstance, taking part in clinical trials offers a great choice for these patients to receive the best therapy with little or no economic burdens. Off-label use of drugs by cancer patients has a long history, especially for those who have little or almost no choice in potentially effective therapy. Because of the high drug costs and the uncertain effectiveness, access to anti-cancer drugs for off-label use is becoming increasingly difficult, and clinical trials may be the only choice. NCCN also indicated in its guidelines that it believes that the best management for any patient is in clinical trials, and participation in clinical trials is especially encouraged. Including clinical trials information in the interpretation report is becoming a necessity. OncoPDSS not only filters the best suitable clinical trials, but also informs patients of the nearest facilities, thus helping patients choose whether to participate.

To the best of our knowledge, OncoPDSS is the first platform that interprets multi-omics alterations into a pharmacotherapy-centric report based on a comprehensive knowledgebase of actionable evidence, drug indications, and clinical trials at the individual level (see Additional file 1, Table S7). Although groundbreaking and effective, there are still limitations. The TScore formula is totally evidence-level and count-based; it only reflects the evidence-supporting status while not the effectiveness. A more precise algorithm needs to be developed and validated to make the platform more significant. To make OncoPDSS a more accurate and valuable clinical decision support system, a more ample knowledgebase with credible evidence is needed as the foundation. In the future, we will continue to integrate and curate more actionable evidence and drug indications and update the information on clinical trials. Drug efficacy and safety are not only influenced by drug targets, as enzymes, transporters and other proteins can also contribute [30, 31]. In addition, drug-drug interactions and even food-drug interactions can change drug responses; therefore, drug ADMET information and drug-drug/food interaction information should be included in the knowledgebase and be taken into account in pharmacotherapy classification.

## Conclusion

OncoPDSS is a practical system that automatically interprets cancer sequencing results as supporting evidence for clinical pharmacotherapy decision making. It receives the alterations detected in cancer samples as input and annotates them with the response changes on related pharmacotherapies based on collected evidence, thus summarizing whether these therapies are potentially useful at the individual level. OncoPDSS also retrieves the most relevant clinical trials that are relevant to the current cancer patient. This platform will greatly benefit both cancer researchers and clinical oncologists to aid in improving their knowledge of oncology pharmacotherapy, providing classification results and plenty of collected evidence, thus helping physicians make their final clinical pharmacotherapy decisions.

### Availability and requirements

Project name: OncoPDSS.

Project home page: https://oncopdss.capitalbiobigdata.com

Operating system(s): Platform independent.

Programming language: Java, Perl.

Other requirements: Java 8 or higher, Tomcat 8.0 or higher.

License: GNU GPL.

Any restrictions to use by non-academics: license needed.

## Supplementary information

**Additional file 1.**

## Data Availability

The tool is freely available through a web interface at https://oncopdss.capitalbiobigdata.com.

## Abbreviations

OncoPDSS: Oncology Pharmacotherapy Decision Support System
FDA: U.S. Food and Drug Administration
NCCN: National Comprehensive Cancer Network
ASCO: American Society of Clinical Oncology
EMSO: European Society for Medical Oncology
APIs: Application Program Interfaces
WGS: Whole Genome Sequencing
WES: Whole Exome Sequencing
NSCLC: Non-Small Cell Lung Cancer
ALK: Anaplastic Lymphoma Kinase
CGI: Cancer Genome Interpreter
PGx: Pharmacogenomics
HUGO: The Human Genome Organisation
HGNC: HUGO Gene Nomenclature Committee
VCF: Variant Call Format
RDBMS: Relational Database Management System
OS: Overall Survival
PFS: Progress Free Survival
