# Supplementary material for: OncoPDSS: an evidence-based clinical decision support system for oncology pharmacotherapy at the individual level
Source: BMC Cancer. 2020 Aug 8;20:740. doi: 10.1186/s12885-020-07221-5 (PMC7414679; doi:10.1186/s12885-020-07221-5)
Supplement: Supplementary file 1 — Additional file 1. [file 12885_2020_7221_MOESM1_ESM.docx]

**Supplementary Data**

**OncoPDSS: an evidence-based clinical decision support system for oncology pharmacotherapy at individual level**

Quan Xu, Jin-Cheng Zhai, Cai-Qin Huo, Yang Li, Ru-Dan Huang, Chuang Shen, Yu-Jun Chang, Xi-Ling Zeng, Fan-Lin Meng, Fang Yang, Yan-Jiao Lu, Hong-Jie Liu, Wan-Ling Zhang, Sheng-Nan Zhang, Yi-Ming Zhou, and Zhi Zhang

**Table of Contents**

**Supplementary Table S1:** Non-Disease Ontology cancer terms. These are the terms that appeared in some of the pharmacotherapy evidences while cannot be exactly matched to the Disease Ontology terms.

| **No.** | **Cancer Identifier** | **Cancer Term** | **Description** |
| --- | --- | --- | --- |
| 1 | NDO:001 | Any Solid Tumor | The therapy/therapies depicted in the evidences can be applied to any solid tumor under certain type of biomarkers. |
| 2 | NDO:002 | Any Cancer Type | The therapy/therapies depicted in the evidences can be applied to any cancer type under certain type of biomarkers. |
| 3 | NDO:003 | Chronic Eosinophilic Leukemia | Chronic eosinophilic leukemia, cannot be exactly matched to Disease Ontology terms. |
| 4 | NDO:004 | Plasmablastic Lymphoma | Plasmablastic lymphoma, cannot be exactly matched to Disease Ontology terms. |
| 5 | NDO:005 | Skin Cancer, Non-Melanoma | Non-melanoma skin cancer, cannot be exactly matched to Disease Ontology terms. |
| 6 | NDO:006 | Advanced Solid Tumor | The therapy/therapies depicted in the evidences can be applied to advanced solid tumor under certain type of biomarkers. |

**Supplementary Table S2:** Alteration types.

| **Category** | **Alteration Type** | **Meaning** | **Examples** |
| --- | --- | --- | --- |
| Genome Level | DEL | Deletion | CHROMOSOME:17p deletion |
|  | DUP | Duplication, including amplications | ERBB2:Amplification |
|  | FS | Frame shift mutation | PTEN:V317fs |
|  | INA | Biallelic Inactivation | UGT1A1:Biallelic Inactivation |
|  | INDEL | Insertion-deletion | ERBB2:G776delinsVC |
|  | INS | Insertion | EGFR:Exon19ins |
|  | MSM | Missense mutation | EGFR:L858R |
|  | MUT | Mutations | KRAS:Mutations |
|  | NSM | Nonsense mutation | KRAS:Q22* |
|  | RAM | Rearrangement, or named gene fusion | ABL1:BCR-ABL |
|  | RAM~DUP | Fusion gene duplication | ALK:EML4-ALK Amplification |
|  | RAM~INS | Fusion gene insertion | ALK:EML4-ALK T1151insT |
|  | RAM~MSM | Fusion gene missense mutation | ALK:EML4-ALK G1269A |
|  | RAM~OTH | Fusion gene other types of mutation | ALK:EML4-ALK E2;A20 |
|  | SNP | Single Nucleotide Polymorphism | MLH1:C.790+1G>A |
|  | WT | Wild-type | BRAF:Wild-type |
| Transcriptome Level* | EXP | Expression | ERBB2:Expression (HER2 positive) |
|  | NEXP | Not expressed, or named negative | ERBB2:Negative (HER2 negative) |
|  | OEXP | Over expression | EGFR:OverExpression (EGFR over expression) |
|  | UEXP | Under expression | BRCA1:UnderExpression (BRCA1 under expression) |
| Epigenome Level | DMET | Demethylation | CCND2:Promoter Demethylation |
|  | EGS | Epigenetic Silencing | CDH1:Epigenetic Silencing |
|  | EGP | Epigenetic Phosphorylation | EGFR:Y1092 Phosphorylation |
|  | HMET | Hypermethylation | PTPRT:Promoter Hypermethylation |
|  | MET | Methylation | MLH1:Methylation |
| Others | AND | Co-alterations | MTOR:E2014K + MTOR:E2419K |
|  | GROUP | Alteration group | EGFR:Activating Mutations |
|  | OR | Any of the mutations | BRAF:V600E/V600K |
|  | OTH | Other unclassified alterations | CHROMOSOME:MSI-H (microsatellite instability-high) |

* This category also contains proteomic level alterations, for example, evidence related to HER2 positive (proteomic based) and evidence related to ERBB2:expression (transcription level) are integrated as the same alteration, and data retrieval is provided according to the latter.

**Supplementary Table S3:** Data sources of OncoPDSS database.

| **Source** | **Actionable**  **Evidence** | **Drug Indications** | **Cancer**  **Clinical Trials** | **Cancer Information** | **Drug Information** | **Alteration Information** | **Gene Information** | **Gene Exon Information** |
| --- | --- | --- | --- | --- | --- | --- | --- | --- |
| CIViC | 2,706 | - | - | - | 321 | - | 334 | - |
| CGI | 1,442 | - | - | - | 109 | - | - | - |
| OncoKB | 237 | - | - | - | - | - | - | - |
| US FDA/Drug Labels | 298 | 526 | - | - | 236 | - | - | - |
| NCCN guidelines | 649 | - | - | - | - | - | - | - |
| ASCO guidelines | 268 | - | - | - | - | - | - | - |
| ClinicalTrials.gov | - | - | 19,922 | - | - | - | - | - |
| DiseaseOntology | - | - | - | 366 | - | - | - | - |
| MeSH | - | - | - | 318 | - | - | - | - |
| DrugBank | - | 15 | - | - | 200 | - | - | - |
| PubChem | - | - | - | - | 1,034 | - | - | - |
| ClinVar | - | - | - | - | - | 555 | - | - |
| COSMIC | - | - | - | - | - | 102 | - | - |
| MyVariant.info | - | - | - | - | - | 10,541 | - | - |
| ANNOVAR | - | - | - | - | - | 10,748 | - | - |
| HGNC | - | - | - | - | - | - | 528 | - |
| Ensembl | - | - | - | - | - | - | - | 1,939 |

**Supplementary Table S4:** Research type standardization.

| **OncoPDSS** | **OncoKB** | **CIViC** | **CGI** | **FDA /**  **Drug Labels** | **NCCN Guidelines** | **ASCO Guidelines** |
| --- | --- | --- | --- | --- | --- | --- |
| Case report | - | Level C (partial) | Case report | - | - | - |
| Clinical trial | Level 3 | Level A (partial); Level B; Level C (partial) | Clinical trials; Early Trials, Case Report; Late trials; Late trials, Pre-clinical; Early trials | - | - | - |
| Approved | Level 1 | Level A (partial) | FDA guidelines | √ | - | - |
| Guideline | Level 2; Level R1 | Level A (partial) | NCCN/CAP guidelines; CPIC guidelines; European LeukemiaNet guidelines; NCCN guidelines | - | √ | √ |
| Inferential | - | Level E | - | - | - | - |
| Pre-clinical | Level 4 | Level D | Pre-clinical | - | - | - |

**Supplementary Table S5:** Clinical significance and guide directions.

| **No.** | **Clinical significance** | **Guide direction** | **Description** |
| --- | --- | --- | --- |
| 1 | Sensitivity or Response or Likely to Benefit | Positive | The current cancer type is sensitive to the pharmacotherapy. |
| 2 | Safe or Well tolerated | Positive | The current pharmacotherapy is safe or well tolerated by the cancer patient(s). |
| 3 | Resistance or Non-Response or Unlikely to Benefit | Negative | The current cancer type is resistant to the pharmacotherapy. |
| 4 | Increased Toxicity | Negative | The toxicity or risk of toxicity is increased for the use of the pharmacotherapy, thus it is not recommended. |
| 5 | Limitation of Use or Uncertain Prognosis or Uncertain Response | Uncertain | The safety or the efficacy of the pharmacotherapy is not clearly described. |

**Supplementary Table S6:** OncoPDSS database (version 1.0) statistics.

| **Dataset** | **Count** | **Dataset** | **Count** |
| --- | --- | --- | --- |
| Alteration-Drug Associations | 7,692 | Anti-Cancer Agents | 2,676 |
| Drug-Indication associations | 526 | Cancer Types | 372 |
| Cancer Clinical Trials | 19,922 | Alterations | 13,889 |
| Clinical Trials-Cancer Associations | 200,546 | Alteration Types | 29 |
| Clinical Trials-Drug Associations | 19,592 | Cancer Pharmacotherapy Genes | 528 |
| Clinical Trials-Gene Associations | 29,484 | Combinatorial Therapy | 579 |
| Clinical Trials-Alteration Associations | 769,817 | Co-Alteration Evidence | 2,012 |
| Clinical Trials-Faculty Associations | 323,600 |  |  |

**Supplementary Table S7:** Comparison of OncoPDSS with other resources.

| Resource | Alterations | System | Interpretation report | Interpretation Center | Drug classification | Drug prioritization score | Clinical trials | FDA drug indications | Data query | Query Input type |
| --- | --- | --- | --- | --- | --- | --- | --- | --- | --- | --- |
| OncoPDSS | Multi-omics | Web-based | √ | Pharmacotherapy | √ | √ | √ | √ | √ | Pharmacotherapy, gene, alteration, cancer, evidence-related |
| CIViC | Multi-omics | Web-based | × | - | - | - | - | - | √ | Gene, alteration, cancer, drug, evidence-related |
| OncoKB | Genomic | Web-based | × | - | - | - | - | - | √ | Gene, cancer, drug |
| PMKB | Genomic | Web-based | × | - | - | - | - | - | √ | Gene, cancer, drug, alteration, interpretation |
| CGI | Multi-omics | Web-based | √ | Alteration | × | × | × | × | √ | Gene, cancer, drug, alteration, evidence-related |
| PanDrugs | Genomic | Web-based | √ | Gene | × | √ | × | × | √ | Gene, drug, alteration |
| mTCTScan | Genomic | Web-based | √ | Drug, alteration | √ | × | √ | × | √ | alteration |
| Personal Cancer Genome Reporter (1) | Genomic | Docker-based | √ | Alteration | × | × | × | × | × | alteration |
| Molecular Tumor Board Report (2) | Genomic | R codes | √ | Alteration | × | × | × | × | × | alteration |
| IMPACT (3) | Genomic | Web-based | √ | Drug | × | × | √ | × | √ | Alteration, gene |

**References:**

1. Nakken S, Fournous G, Vodak D, Aasheim LB, Myklebost O, Hovig E. Personal Cancer Genome Reporter: variant interpretation report for precision oncology. Bioinformatics. 2018;34(10):1778-80.

2. Perera-Bel J, Hutter B, Heining C, Bleckmann A, Frohlich M, Frohling S, et al. From somatic variants towards precision oncology: Evidence-driven reporting of treatment options in molecular tumor boards. Genome Med. 2018;10(1):18.

3. Hintzsche JD, Yoo M, Kim J, Amato CM, Robinson WA, Tan AC. IMPACT web portal: oncology database integrating molecular profiles with actionable therapeutics. BMC Med Genomics. 2018;11(Suppl 2):26.

**Supplement on TScore equation**

TScore = $\sum_{i=1}^{n} \left（ E_{i}+S_{di}*C_{di} \right）$ + lg(*C_ct_* + 1)

As we have described in the manuscript, the consideration of a pharmacotherapy is related to the relevant actionable evidence, drug indications, and clinical trials. All actionable evidence can be classified into six categories, namely approved drug labels, guidelines, clinical trials, case reports, pre-clinical information, and inferential procedures according to their initial sources, and each can be assigned a confidence level score. The drug indication evidence is the medication information related to specific cancer species, and does not involve specific omics characteristics (in the OncoPDSS database). Although it is approved evidence, it is not targeted at the population with specific omics characteristics. Therefore, the evidence level is not equal to the approved actionable evidence, and the score is naturally set to be smaller. The number of clinical trials is only to help users make a better choice from another dimension when the evidence level of the pharmacotherapy is similar. It is not an absolute standard to judge whether a pharmacotherapy is good or not. Therefore, its impact on TScore should be relatively small, which can distinguish the pharmacotherapy with the same evidence score, and not have a significant impact on the global ranking. It must be noted that the TScore does not directly reflect the effectiveness prioritization and is only one of the many ways to sort the pharmacotherapies.
